# High-Protein, Low-Glycaemic Meal Replacement Improves Physical Health-Related Quality of Life in High-Risk Persons for Metabolic Syndrome—A Subanalysis of the Randomised-Controlled ACOORH Trial

**DOI:** 10.3390/nu14153161

**Published:** 2022-07-30

**Authors:** Kerstin Kempf, Martin Röhling, Winfried Banzer, Klaus Michael Braumann, Martin Halle, Nina Schaller, David McCarthy, Hans Georg Predel, Isabelle Schenkenberger, Susanne Tan, Hermann Toplak, Stephan Martin, Aloys Berg, on behalf of the ACOORH Study Group

**Affiliations:** 1 West-German Centre of Diabetes and Health, Düsseldorf Catholic Hospital Group, Hohensandweg 37, 40591 Düsseldorf, Germany; martin.roehling@vkkd-kliniken.de (M.R.); stephan.martin@vkkd-kliniken.de (S.M.); 2 Institute for Sports and Sport Science, Department of Sports Medicine, University of Frankfurt, 60487 Frankfurt, Germany; banzer@med.uni-frankfurt.de; 3 Department of Sports and Movement Medicine, Faculty of Psychology and Human Movement Sciences, University of Hamburg, 20148 Hamburg, Germany; braumann@uni-hamburg.de; 4 Department of Prevention, Rehabilitation and Sports Medicine, Klinikum Rechts der Isar, Technical University of Munich (TUM), 80992 Munich, Germany; martin.halle@mri.tum.de (M.H.); nina.schaller@mri.tum.de (N.S.); 5 DZHK (German Centre for Cardiovascular Research), Partner Site Munich Heart Alliance, 80992 Munich, Germany; 6 Public Health Nutrition Research Group, London Metropolitan University, London N7 8DB, UK; hdavid.mccarthy@gmail.com; 7 Institute of Cardiovascular Research and Sports Medicine, German Sport University Cologne, 50933 Cologne, Germany; predel@dshs-koeln.de; 8 KARDIOS, Cardiologists in Berlin, 10787 Berlin, Germany; schenkenberger@klinische-forschung-berlin.de; 9 Department of Endocrinology, Diabetes and Metabolism and Division of Laboratory Research, University Hospital Essen, University Duisburg-Essen, 45122 Essen, Germany; susanne.tan@uk-essen.de; 10 Department of Medicine, Division of Endocrinology, Medical University of Graz, 8010 Graz, Austria; hermann.toplak@medunigraz.at; 11 Faculty of Medicine, Heinrich Heine University Düsseldorf, 40591 Düsseldorf, Germany; 12 Faculty of Medicine, University of Freiburg, 79117 Freiburg, Germany; berg.aloys@web.de

**Keywords:** health-related quality of life, protein-rich, low-glycaemic meal replacement, weight reduction, multicenter study, RCT

## Abstract

While obesity impairs health-related quality of life (HRQOL), lifestyle interventions targeting weight reduction have been effective in improving HRQOL. Therefore, we hypothesised that a meal replacement-based lifestyle intervention, which has been shown to successfully reduce weight, would also improve HRQOL more effectively than a lifestyle intervention alone. In the international, multicenter, randomised-controlled ACOORH-trial (Almased-Concept-against- Overweight-and-Obesity-and-Related-Health-Risk), overweight or obese participants with elevated risk for metabolic syndrome (*n* = 463) were randomised into two groups. Both groups received telemonitoring devices and nutritional advice. The intervention group additionally used a protein-rich, low-glycaemic meal replacement for 6 months. HRQOL was estimated at baseline, after 3 and 12 months, using the SF-36 questionnaire, and all datasets providing HRQOL data (*n* = 263) were included in this predefined subanalysis. Stronger improvements in the physical component summary (PCS) were observed in the intervention compared to the control group, peaking after 3 months (estimated treatment difference 2.7 [1.2; 4.2]; *p* < 0.0001), but also in the long-term. Multiple regression analysis demonstrated that insulin levels and the achieved weight loss were associated with the mental component summary (MCS) after 12 months (*p* < 0.05). Thus, meal replacement-based lifestyle intervention is not only effective in weight reduction but, concomitantly, in enhancing HRQOL.

## 1. Introduction

The classification of health-related quality of life (HRQOL) consists of self-perceived physical and mental well-being [1]. Obesity not only negatively impacts HRQOL [2], it also increases the risk of other diseases such as metabolic syndrome, which in turn further worsens HRQOL [3].

In Germany, about 20% of residents are affected by metabolic syndrome [4], which is a combination of abdominal obesity, hyperglycemia, hypertriglyceridemia, low levels of high-density lipoprotein (HDL)-cholesterol, and hypertension. Although each of these risk factors has been shown to be influenceable by structured lifestyle interventions, drug therapy is overwhelmingly used [5]. It seems to be easier for the affected to take medication in order to improve blood glucose, blood lipids, or blood pressure rather than changing their lifestyle.

To find a way to change the lifestyle of overweight or obese people at increased risk of metabolic syndrome, the international, multicenter ALMASED-Concept-against-Overweight-and-Obesity-and-Related-Health-Risk (ACOORH) study [6,7,8,9,10,11] was initiated. With this randomised-controlled trial, the effects of a meal replacement-based lifestyle intervention vs. lifestyle intervention alone were compared, and it was shown that a significantly higher weight reduction occurred in the meal replacement intervention group compared to the control group [6], accompanied by improvements of further risk factors [7,8,10], predominantly the reduction of hyperinsulinemia [9] and hyperleptinemia [11]. However, the best-designed lifestyle intervention is useless if the participants are not comfortable with it. Due to individual barriers, or if participants do not see further benefit for their HRQOL in addition to the weight reduction, the lifestyle alteration might not be integrated into everyday life in the long term [12].

Therefore, in this predefined sub-analysis of the ACOORH trial, we analysed the long-term effects of a meal replacement intervention on the physical and mental dimensions of HRQOL.

## 2. Materials and Methods

### 2.1. Study Design and Population

The effect of a high-protein, low-glycaemic meal-replacement on HRQOL was analysed in the international, multicenter, randomised-controlled ACOORH trial, which included overweight or obese persons with components of the metabolic syndrome. The study design and further details have been published before [6,7,8,9,10,11]. In brief, individuals with a body mass index (BMI) of 27–35 kg/m^2^ and/or a waist circumference of ≥88 (females) or ≥102 cm (males), 21–65 years old, who had of least one of the following criteria of the metabolic syndrome: (a) fasting blood glucose (FBG) 100–125 mg/dL, (b) triglycerides 150–400 mg/dL, (c) HDL cholesterol < 40 mg/dL, or (d) untreated systolic blood pressure of 140–160 mmHg or diastolic blood pressure of 90–100 mmHg or anti-hypertensive medication were eligible for participation. Exclusion criteria were (i) diabetes mellitus with FBG ≥ 126 mg/dL or HbA1c ≥ 6.5% (≥48 mmol/mol) or diabetes-related medical history (e.g., antidiabetic drugs or medical records); (ii) total body weight >141 kg; (iii) acute infections; (iv) chronic diseases such as cancer, chronic obstructive pulmonary disease, asthma, chronic gut diseases, nephropathy, and kidney insufficiency with glomerular filtration rate < 30 mL/min/1.73 m^2^, liver cirrhosis, psychoses, dementia, (v) plans to move to areas unserved by ACOORH; (vi) (planned) smoking cessation during the study phase; (vii) medication for active weight reduction; (viii) pregnancy or breast-feeding; and (ix) known intolerance with components of the used meal replacement. The study was conducted in accordance with the Declaration of Helsinki, approved by the responsible ethics committees of all participating centres, with the primary ethical approval obtained from the ethic commission of the Albert-Ludwigs-University Freiburg (approval code 216/14), and registered at drks.de (no. DRKS00006811). Participants were randomised (allocation ratio 1:2) into either a control group or an intervention group with meal replacement-based lifestyle. The first participant was included in January 2015 and the last examination was performed in August 2017. The assessors were blinded for group allocation. The initial ACOORH cohort included 463 participants. In the present predefined subanalysis, only those with at least two datasets for HRQOL (*n* = 263) were considered.

### 2.2. Intervention and Meal Replacement Regimen

Participants of both groups visited the study centre at baseline, as well as after 1, 3, 6, and 12 months. They received nutritional counselling and guidance on increasing physical activity and were equipped with pedometers and telemetric scales [6]. Collected data (i.e., body weight, steps) were automatically transferred into a personalised online portal and discussed during study visits. Target agreements were fixed, and participants were motivated to achieve their individual goals (e.g., weight reduction, steps, healthy lifestyle changes). The intervention group additionally received a high-protein, low-glycaemic meal replacement (Almased; Almased Wellness GmbH, Oberding, Germany). A 6-month intensive meal-replacement phase was followed by a follow-up phase until month 12 [6]. Thus, in the first week, participants were instructed to replace all three main meals. In the 2nd–4th week, breakfast and dinner were replaced, and from the 5th–26th week, dinner was replaced. Detailed information on how to prepare the meal replacement, as well as general nutritional information focussing on the effects of carbohydrate reduction, was provided in an accompanying manual.

### 2.3. Outcomes and Measurements

HRQOL was queried at baseline, as well as after 3 and 12 months. During the study visit, participants filled in the validated self-reporting questionnaire ‘36-Item Short-Form Health Survey’ (SF-36) [1]. It consists of 36 items that determine four physical and four mental dimensions, which are additionally combined as physical component summary (PCS) and mental component summary (MCS). Each dimension can reach a score from 0 to 100, and the HRQOL is higher the higher the score is. Anthropometric data (e.g., body weight, BMI) and blood were obtained and analysed as described before [6,7,8,9,10]. Adverse and serious adverse events were continuously reviewed by an external monitor [6].

### 2.4. Statistics

Sample size calculation can be found elsewhere [6]. If not otherwise stated, intention-to-treat (ITT) analyses were performed and missing values were imputed using the ‘last-observation-carried-forward’ (LOCF) principle. In this predefined subanalysis, the tertiary outcome, the estimated treatment difference (ETD) of HRQOL after 3 and 12 months, was compared between groups. An independent institute (ACOMED Statistik^®^, Leipzig, Germany), which was not involved in the study execution, performed the basic statistical analysis. SPSS 22.0 (SPSS Inc., Chicago, IL, USA) and GraphPad Prism 6.04 (GraphPad Software, San Diego, CA, USA) were used for statistical analysis. Non-parametric data were analysed with the Mann–Whitney test, Wilcoxon signed-rank test, Friedman test with Dunn’s multiple comparison test, or Spearman correlation. Hedges’ g effect size and 95% confidence intervals (CIs) were calculated for each physical and mental dimension measured by SF-36 [13]. Multivariable linear regression analyses were performed to examine the associations between changes in HRQOL with body weight or weight changes, adjusting for potential confounders. Two-sided statistical tests were used, and the level of significance was *p* = 0.05.

## 3. Results

### 3.1. Overall Increase in PCS Items

**Figure 1:**
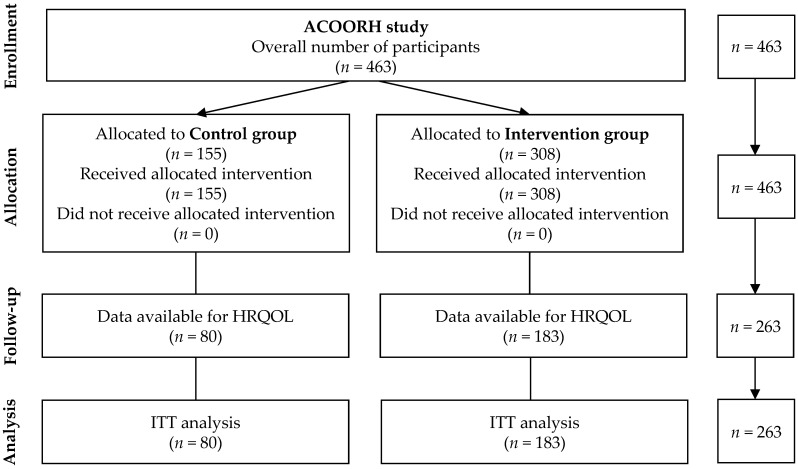
Flow chart. ACOORH: Almased Concept against Overweight and Obesity and Related Health Risk; HRQOL: health-related quality of life; ITT: intention-to-treat.

HRQOL data were available from 80 participants of the control group and from 183 participants of the intervention group. These data were used for ITT-analysis (Figure 1). Age and proportion of female and male did not significantly differ between the participants that were included in the analysis (*n* = 263) and those not included in the analysis (*n* = 200). However, the analysed participants had lower values for weight and BMI (Supplemental Table S1).

Baseline characteristics (Table 1) did not differ significantly between groups, with the exception of higher values for BMI and weight in the control group.

**Figure 2:**
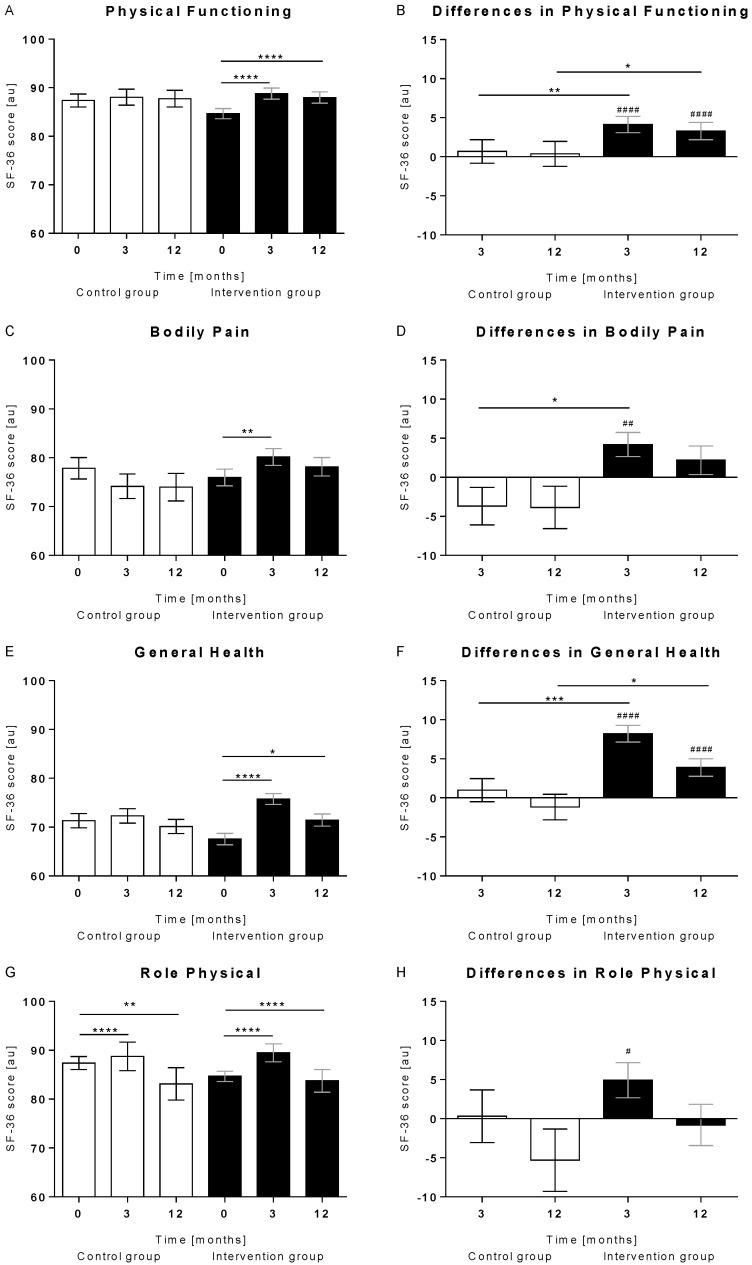
Physical components summary (PCS) dimensions. Shown are absolute and Δ values of the four PCS dimensions (**A**,**B**) physical functioning, (**C**,**D**) bodily pain, (**E**,**F**) general health, and (**G**,**H**) role physical in the control (*n* = 80) and the intervention (*n* = 183) group. Data were analysed with the Friedman test with Dunn’s multiple comparison test (*, *p* < 0.05; **, *p* < 0.01; ***, *p* < 0.001; ****, *p* < 0.0001). Within-group differences were compared using the Wilcoxon signed-rank test (^#^, *p* < 0.05; ^##^, *p* < 0.01; ^####^, *p* < 0.0001) and between group differences using the Mann-Whitney test (*, *p*< 0.05; **, *p* < 0.01; ***, *p* < 0.001). Data are shown as mean ± standard error of means. au, arbitrary units.

The four PCS dimensions—physical functioning, bodily pain, general health, and role-physical—significantly improved in the intervention group within 3 months of intervention (Figure 2), whereas out of the four MCS dimensions, only a significant improvement for vitality (*p* < 0.0001) was observed (data not shown). With the exception for role-physical, no statistically significant alteration occurred in the control group.

### 3.2. Stronger Improvement of the Physical Component Score in the Intervention Group

**Figure 3:**
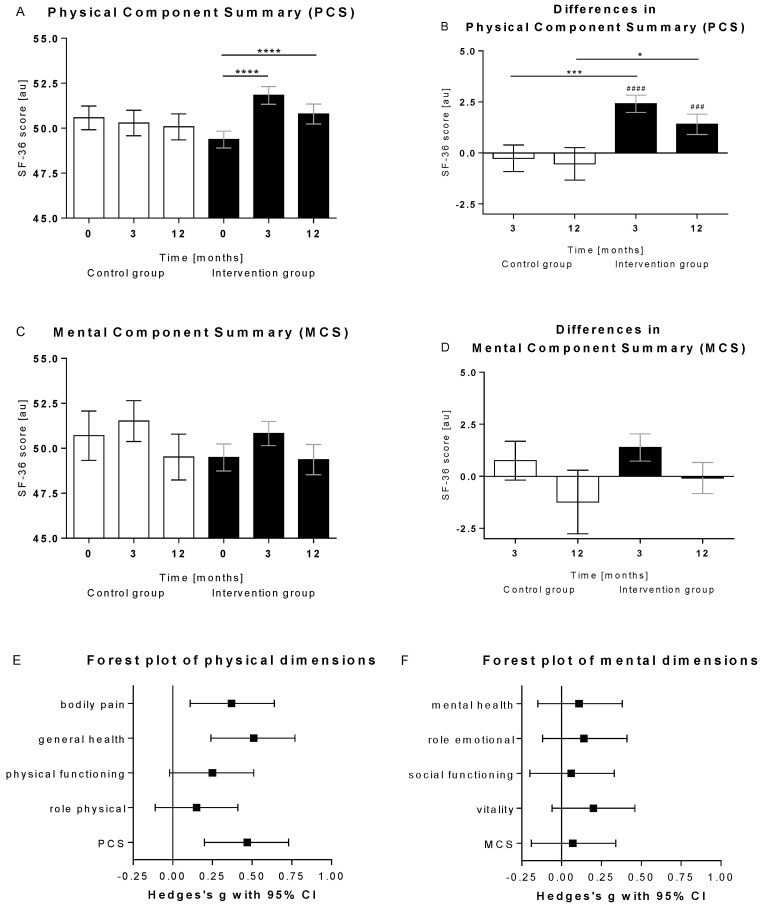
Physical and mental component summary. Shown are (**A**) absolute and (**B**) Δ values of the physical component score (PCS) in the control (*n* = 80) and the intervention (*n* = 183) group at baseline as well as after 3 months of intervention and after 12 months (=6 months after end of intervention). (**C**) shows absolute and (**D**) Δ values of the mental component score (MCS). Data were analysed with the Friedman test and with Dunn’s multiple comparison test (****, *p* < 0.0001). Within-group differences were compared using the Wilcoxon signed-rank test (^###^, *p* < 0.001; ^####^, *p* < 0.0001) and between group differences using the Mann–Whitney test (*, *p*< 0.05; ***, *p* < 0.001). Data are shown as mean ± standard error of means. au, arbitrary units. Forest plot of (**E**) physical and (**F**) mental dimensions. Black squares symbolise the Hedges’s g point estimate of the effect size and the horizontal lines represent the 95% confidence intervals (CI).

During the intervention, PCS only increased in the intervention group, whereas in the control group, PCS rather tended to decrease. Peak values were seen after 3 months (*p* < 0.0001), but also, after 12 months, values were significantly higher than baseline (*p* < 0.0001). The changes in PCS were significantly different between groups, with an ETD of 2.7 [1.2; 4.2] (*p* < 0.0001) after 3 months and of 1.9 [0.3; 3.8] (*p* = 0.021) after 12 months (Figure 3A,B). Contrary to PCS, MCS courses tended to increase during intervention, although changes did not reach statistical significance (*p* = 0.201 in the control and *p* = 0.055 in the intervention group; Figure 3C,D).

### 3.3. Weight Reduction Is Predictive for Improvements in MCS after 12 Months

In order to discover parameters with a general predictive value for long-term improvements in PCS or MCS, the data of the control and the intervention group have been combined (Table 2).

Multivariate linear regression analyses with adjustment to potential confounders such as group, sex, age, BMI, PCS, and MCS at baseline demonstrated a significant association of Δ weight after 12 months with PCS and Δ PCS as well as MCS and Δ MCS after 12 months. Moreover, insulin levels at baseline were predictive for PCS and Δ PCS after 12 months, and insulin levels after 3 months predicted MCS and Δ MCS after 12 months. Changes in leptin after 3 months were associated with PCS and Δ PCS after 12 months, and changes in leptin after 12 months were associated with MCS and Δ MCS after 12 months.

The fully adjusted model demonstrated an independent predictive value of Δ weight after 12 months (*p* = 0.038) and insulin levels after 3 months (*p* = 0.007) for MCS after 12 months (Table 3), i.e., the lower the insulin levels during the intervention and the higher the weight loss after 12 months, the better the mental well-being.

**Figure 4:**
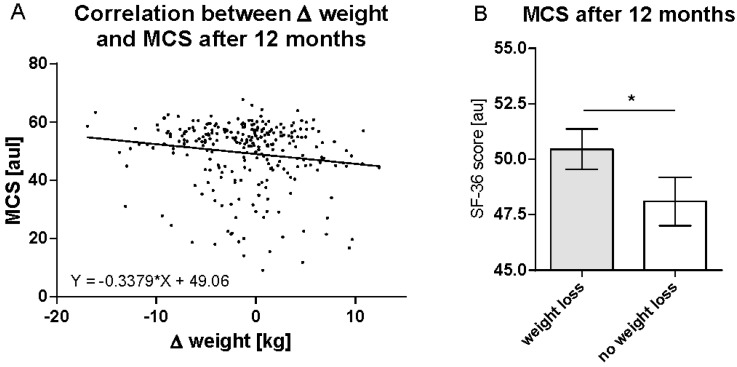
Weight loss accounts for MCS after 12 months. Shown is (**A**) the correlation between Δ weight and mental component summary (MCS) after 12 months (*n* = 263). Linear regression analysis was performed. (**B**) MCS after 12 months is shown after stratifying the cohort into two groups (*n* = 147 vs. 116) according to weight loss. Differences were analysed using the Mann–Whitney test (*, *p* < 0.05). au, arbitrary units.

Weight change accounts for MCS after 12 months. When the weight remained unchanged during the study period, the mean MCS value after 12 months was 49.06. With each kilogram of weight lost, MCS increased by 0.3379 (Figure 4A). Stratification of the cohort (independent of their affiliation to control or intervention group) by weight loss demonstrated significantly higher MCS values after 12 months for those participants who maintained weight loss until follow-up (MCS 50.5 ± 11.0) vs. those who showed no weight loss or weight regain (MCS 48.1 ± 11.8; *p* = 0.47; Figure 4B).

## 4. Discussion

In the international, multicentre, randomised-controlled ACOORH trial, a high-protein, low-glycaemic, meal replacement-based lifestyle intervention was more effective in improving HRQOL than a control lifestyle intervention alone. The improvement of PCS and all individual items of the PCS were significantly more pronounced in the intervention group, whereas higher weight loss was generally associated with higher values of MCS.

So far, we can demonstrate that starting a lifestyle intervention accompanied by high-protein, low-glycaemic meal replacement for overweight or obese persons with at least one criterion of the metabolic syndrome reduced body weight more effectively compared to controls with an ETD of −3.2 kg [−4.0; −2.5] and was able to maintain weight loss long-term [6]. These effects were accompanied by significantly stronger improvements in blood pressure [10]. Individuals with prediabetes also benefited more, since significantly more participants of the intervention group (50 vs. 31%) converted to normoglycemia [7]. Meal-replacement and accompanying nutrition counselling also seem to have influenced nutritional behaviour long term, since there was a decrease in daily carbohydrate consumption but a significant increase in dietary protein in the intervention group [8]. Moreover, significantly higher reductions in insulin, leptin, and inflammation markers were observed, which might explain why it was easier for the participants of the intervention group to lose weight [9,11]. However, all those improvements in clinical and laboratory parameters would be useless if participants would feel worse during the intervention, and replacing meals with a liquid meal replacement might have the potential to negatively impact quality of life.

However, having to go without solid food and using meal replacement for a period of time does not appear to negatively affect patients’ well-being, since the improvements in PCS and MCS were higher during the meal replacement phase compared to the follow -up phase. Participants seem to be able to endure the meal replacement well if they experience weight loss in return [14,15]. A current meta-analysis of Marcos-Delgado et al. [16] has shown that lifestyle interventions have the potential to improve HRQOL, but not all types of intervention appear to be equally effective. With Hedges’ g [95% confidence interval] standardised effect size of 0.47 [0.20; 0.73], our results for the overall effect in physical dimensions were perfectly in line with the reported meta-analysis data of 0.60 [0.31; 0.88], not only with respect to the overall effect size in PCS of but also with the expected effects on the individual dimensions. Thus, the strongest improvements can be seen for general health, followed by bodily pain, and the lowest effects for physical functioning and role-physical. Thus, our findings confirm earlier reports demonstrating beneficial effects of the used meal replacement on HRQOL measured by SF-36. In obese females as well as in overweight type 2 diabetes, patients’ weight loss and increase in HRQOL were more pronounced in the groups with the meal replacement-based lifestyle intervention [17,18]. In the latter, the observed effects on HRQOL were stronger for the PCS than for MCS, too [18]. Therefore, it could be speculated that bioactive components of the meal replacement might positively affect physical dimensions of HRQOL [19].

The connection between weight loss achieved and the improvement in MCS seems easy to explain. The predictive value of lower insulin levels (after 3 months) for the higher mental HRQOL after 12 months is less obvious. However, its potential role in the pathogenesis of depression [20] and the positive association of hyperinsulinemia with depression [21] support the correlation between lower insulin levels and higher MCS. In addition, we were able to show in advance that low insulin levels predict a higher success in weight loss [9]. Therefore, the insulin levels are closely linked to the change of weight. Although conflicting results have been reported for the correlation between leptin and depression [22], lifestyle intervention programs have shown that decreasing leptin levels predicted amelioration in depression symptoms independent of changes in body mass or fat mass [23] and improvement of MCS in metabolic syndrome [24].

Several limitations and strengths of our analysis need to be mentioned. As always with the analysis of questionnaires, it can be pointed out that the data are based on self-reports and were not measured objectively. However, the SF-36 is the most commonly used and one of the best-validated questionnaires for determining HRQOL [16] and is particularly useful in comparing HRQOL across diseases, health conditions, or populations. Thus, the results might be meaningful for people at increased risk for the metabolic syndrome, and obesity-specific quality-of-life measures might be particularly useful, e.g., for prediction of long-term compliance. Conducting research applying such obesity-specific HRQOL measures to examine further the meal replacement-based lifestyle intervention’s health effects might be promising in the future.

The results might further be limited by the fact that HRQOL data have been available from only 263 out of 463 participants. One explanation for this high missing rate is that the SF-36 questionnaire has only been used in the study centres in Germany and Austria. Additionally, SF-36 data were missing from participants who dropped out and did not come to the final study visit. Indeed, it can be assumed that those people who could not keep up with the lifestyle intervention were more likely to drop out early; therefore, the study results could have been biased and the improvement in quality of life overestimated. However, since the dropout rate in the control group was higher than in the intervention group, this would rather lead to an underestimation of the estimated treatment difference. Another criticism could be that individual missing values were replaced by LOCF. This conservative type of imputation method basically assumes that there are minimal changes over time for the missing data. Thus, the analysis might have led to biased and rather underestimated within-group differences. However, since the number of missing values was comparable in both groups, the statements on the group differences should not be distorted.

Higher values for BMI and weight have been observed in the control group. A higher BMI is usually associated with a lower HRQOL [16]. However, the baseline values for PCS and MCS were not statistically significantly different between the groups and rather showed a trend towards lower baseline values in the intervention group. It can therefore be assumed that the higher values for BMI and weight in the control group at baseline have not had any significant influence on the results.

An additional bias might have occurred in the correlation analyses when the participants in the control and intervention groups were analysed together. However, this was due to the fact that the number of participants in separated groups would have been too small to be able to find statistically significant associations. In addition, this analysis was intended to examine general effects for which group membership plays a subordinate role. A strength of our work is that we analysed the influence of BMI and weight change in a multivariate regression model, along with other factors such as baseline PCS and MCS. Although the absolute values of PCS increased significantly only in the intervention group and a significant difference in the change in the PCS after 12 months has been observed between the groups, correlation analysis did not show a significant impact for group membership. This is explained by the fact that the absolute PCS level after 12 months was mainly dependent on the absolute PCS value at baseline, which tended to be slightly lower in the intervention group at baseline. Since, also, BMI has been shown to have a negative impact on HRQOL, it was rightly demanded [16] that these important parameters have to be taken into account when determining the effectiveness of an intervention.

## 5. Conclusions

In the ACOORH trial, the addition of a high-protein, low-glycaemic meal replacement led to a significantly stronger improvement in physical components of HRQOL than lifestyle intervention alone. Moreover, higher weight loss accounts for stronger improvements in mental components of HRQOL.

## Figures and Tables

**Table 1:** Baseline characteristics.

Parameters	Control Group (*n* = 80)	Intervention Group (*n* = 183)
Sex [%] female/male	57.5/42.5	65.0/35.0
Age [years]	50.1 ± 9.8	51.5 ± 9.0
Body mass index [kg/m^2^]	30.8 ± 2.3	29.9 ± 2.3 *
Weight [kg]	92.2 ± 12.5	87.2 ± 12.7 **
PCS [au]	50.6 ± 5.9	49.4 ± 6.4
MCS [au]	50.7 ± 12.2	49.5 ± 10.1
Fasting insulin [µU/mL]	14.1 ± 7.2	13.4 ± 8.6
Leptin [µg/L]	13.5 ± 9.5	14.6 ± 10.3

Shown are mean ± standard deviation or median [interquartile range]. Fisher’s exact test and the Mann–Whitney test were used for comparisons between groups (*, *p* < 0.05; **, *p* < 0.01). PCS, physical component summary; MCS, mental component summary.

**Table 2:** Associations between PCS and MCS with weight, insulin, and leptin.

	PCS after 12 Months				Δ PCS after 12 Months			
Parameters	*r*	*p*	*ß*	*p ^#^*	*r*	*p*	*ß*	*p ^#^*
weight (baseline)	0.012	0.847	−0.065	0.464	−0.056	0.362	−0.68	0.465
weight (3 months)	−0.023	0.711	−0.117	0.186	−0.074	0.230	−0.121	0.186
weight (12 months)	−0.040	0.519	−0.141	0.085	−0.088	0.155	−0.145	0.085
Δ weight (3 months)	−0.178	**0.004**	−0.102	0.065	−0.090	0.145	−0.105	0.065
Δ weight (12 months)	−0.096	0.120	−0.113	**0.040**	−0.067	0.277	−0.117	**0.040**
insulin (baseline)	−0.107	0.084	−0.140	**0.018**	−0.052	0.402	−0.145	**0.018**
insulin (3 months)	−0.058	0.346	−0.72	0.195	−0.036	0.557	−0.075	0.196
insulin (12 months)	−0.055	0.372	−0.53	0.353	−0.020	0.749	−0.055	0.352
Δ insulin (3 months)	0.036	0.566	0.041	0.453	0.027	0.664	0.043	0.453
Δ insulin (12 months)	0.011	0.858	0.059	0.295	0.001	0.987	0.060	0.296
leptin (baseline)	−0.038	0.536	0.081	0.234	0.099	0.111	0.084	0.234
leptin (3 months)	−0.181	**0.003**	−0.110	0.097	0.000	0.995	−0.114	0.097
leptin (12 months)	−0.131	**0.033**	−0.014	0.828	0.046	0.462	−0.014	0.828
Δ leptin (3 months)	−0.207	**0.001**	−0.136	**0.015**	−0.191	**0.002**	−0.141	**0.015**
Δ leptin (12 months)	−0.127	**0.041**	−0.060	0.281	−0.033	0.591	−0.062	0.281
	**MCS after 12 months**				**Δ MCS after 12 months**			
**Parameters**	***r***	***p***	***ß***	***p ^#^***	***r***	***p***	***ß***	***p ^#^***
weight (baseline)	0.093	0.132	0.118	0.176	−0.079	0.203	0.119	0.176
weight (3 months)	0.079	0.203	0.068	0.427	−0.115	0.064	0.069	0.427
weight (12 months)	0.017	0.778	−0.037	0.642	−0.141	**0.022**	−0.038	0.642
Δ weight (3 months)	−0.065	0.296	−0.085	0.117	−0.202	**0.001**	−0.086	0.117
Δ weight (12 months)	−0.148	**0.016**	−0.161	**0.003**	−0.198	**0.001**	−0.163	**0.003**
insulin (baseline)	−0.104	0.093	−0.068	0.239	−0.144	**0.020**	−0.069	0.239
insulin (3 months)	−0.132	**0.033**	−0.142	**0.009**	−0.238	**<0.001**	−0.144	**0.009**
insulin (12 months)	−0.062	0.314	−0.098	0.082	−0.161	**0.009**	−0.099	0.082
Δ insulin (3 months)	−0.036	0.557	−0.087	0.107	−0.135	**0.028**	−0.088	0.108
Δ insulin (12 months)	0.066	0.287	−0.036	0.515	−0.010	0.878	−0.036	0.515
leptin (baseline)	−0.088	0.157	0.074	0.271	0.060	0.334	0.074	0.271
leptin (3 months)	−0.122	**0.049**	0.000	0.998	−0.045	0.472	0.000	0.998
leptin (12 months)	−0.194	**0.002**	−0.108	0.079	−0.117	0.058	−0.110	0.079
Δ leptin (3 months)	−0.007	0.913	−0.056	0.310	−0.101	0.101	−0.056	0.310
Δ leptin (12 months)	−0.117	0.059	−0.137	**0.012**	−0.144	**0.020**	−0.138	**0.012**

*n* = 263 datasets have been analysed. Bold *p*-values represent statistical significance. Spearman correlation and multivariate linear regression analyses ^#^ adjusted to group, sex, age, body mass index (BMI), physical component score (PCS), and mental component score (MCS) at baseline were performed. The grey-marked parameters were used for the adjustment in the next model.

**Table 3:** Predictive parameters for improvement in PCS or MCS after 12 months.

	PCS after 12 Months				MCS after 12 Months			
Parameters	*r*	*p*	*ß*	*p ^#^*	*r*	*p*	*ß*	*p ^#^*
Group	0.094	0.130	0.084	0.136	−0.026	0.679	0.045	0.402
Sex	−0.133	**0.031**	−0.060	0.298	−0.126	**0.042**	−0.043	0.431
Age	−0.194	**0.002**	−0.095	0.095	0.139	**0.024**	0.035	0.521
BMI (at baseline)	−0.123	**0.046**	0.016	0.791	−0.080	0.198	−0.023	0.675
PCS (at baseline)	0.464	**<0.001**	0.444	**<0.001**	0.056	0.368	0.155	**0.006**
MCS (at baseline)	−0.094	0.127	0.043	0.433	0.486	**<0.001**	0.491	**<0.001**
Δ weight	−0.096	0.120	−0.064	0.268	−0.148	**0.016**	−0.123	**0.038**
insulin ^a^	−0.107	0.084	−0.116	0.052	−0.132	**0.033**	−0.145	**0.007**
Δ leptin ^b^	−0.207	**0.001**	−0.103	0.080	−0.117	0.059	−0.077	0.199

*n* = 263 datasets have been analysed. Bold *p*-values represent statistical significance. Spearman correlation and multivariate linear regression analyses ^#^ adjusted to group, sex, age, body mass index (BMI) at baseline, physical component score (PCS) at baseline, mental component score (MCS) at baseline, and Δ weight after 12 months, insulin ^a^ at baseline (for PCS) and after 3 months (for MCS), and Δ leptin ^b^ after 3 months (for PCS) and after 12 months (for MCS) were performed.

## Data Availability

The datasets generated during and/or analysed during the current study are not publicly available but are available from the corresponding author on reasonable request.

## Supplementary Materials

The following are available online at https://www.mdpi.com/article/10.3390/nu14153161/s1, Table S1: Baseline characteristics of participants with and without HRQOL data.
