# Supplementary material for: High-Protein, Low-Glycaemic Meal Replacement Improves Physical Health-Related Quality of Life in High-Risk Persons for Metabolic Syndrome—A Subanalysis of the Randomised-Controlled ACOORH Trial
Source: Nutrients. 2022 Jul 30;14(15):3161. doi: 10.3390/nu14153161 (PMC9370463; doi:10.3390/nu14153161)
Supplement: Supplementary file 1 [file nutrients-14-03161-s001.zip › nutrients-1846355-supplementary.pdf]

Supplemental Table S1. Baseline characteristics of participants with and without HRQOL data.

**Parameters**	**Participants with HRQOL data (*n* = 263)**	**Participants without HRQOL data (*n* = 200)**
Sex [%] female/male	62.7 / 37.3	66.5 / 33.5
Age [years]	51.1 ± 9.2	49.5 ± 10.0
Body mass index [kg/m^2]	30.2 ± 2.3	31.8 ± 2.4****
Weight [kg]	88.7 ± 12.8	92.7 ± 13.1**

Shown are mean ± standard deviation. Fisher's exact and Mann Whitney test were used for comparisons between groups (**, p <0.01; ****, p < 0.0001). HRQOL, health-related quality of life.
